# Building-Level Detection Threshold of SARS-CoV-2 in Wastewater

**DOI:** 10.1128/spectrum.02929-22

**Published:** 2023-03-28

**Authors:** Nicole C. Rondeau, Oliver J. Rose, Ellen R. Alt, Lina A. Ariyan, Annabelle B. Elikan, Jenna L. Everard, Abigail R. Schreier, Maya E. Tessler, Grace H. Tulinsky, Janet R. Vo, Caroline A. Ray, Cynthia Y. Yang, JJ L. Miranda, Brian J. Mailloux

**Affiliations:** a Department of Biology, Barnard College, Columbia University, New York, New York, USA; b Office of Facilities Services, Barnard College, Columbia University, New York, New York, USA; c Environmental Science Department, Barnard College, Columbia University, New York, New York, USA; d President’s Office, Barnard College, Columbia University, New York, New York, USA; University of São Paulo

**Keywords:** SARS-CoV-2, epidemiology, wastewater

## Abstract

We established wastewater surveillance of SARS-CoV-2 in a small, residential, urban college as part of an integrated public health response during the COVID-19 pandemic. Students returned to campus in spring 2021. During the semester, students were required to perform nasal PCR tests twice weekly. At the same time, wastewater monitoring was established in 3 campus dormitory buildings. Two were dedicated dormitories with populations of 188 and 138 students; 1 was an isolation building where students were moved within 2 h of receiving positive test results. Analysis of wastewater from isolation indicated that the amount of viral shedding was highly variable and that viral concentration could not be used to estimate the number of cases at the building level. However, rapid movement of students to isolation enabled determination of predictive power, specificity, and sensitivity from instances in which generally one positive case at a time occurred in a building. Our assay yields effective results with an ~60% positive predictive power, ~90% negative predictive power, and ~90% specificity. Sensitivity, however, is low at ~40%. Detection is improved in the few instances of 2 simultaneous positive cases, with sensitivity of 1 case versus 2 cases increasing from ~20% to 100%. We also measured the appearance of a variant of concern on campus and noted a similarity in timeline with increased prevalence in surrounding New York City. Monitoring SARS-CoV-2 in the sewage outflow of individual buildings can be used with a realistic goal of containing outbreak clusters but not necessarily single cases.

**IMPORTANCE** Diagnostic testing of sewage can detect levels of circulating viruses to help inform public health. Wastewater-based epidemiology has been particularly active during the COVID-19 pandemic to measure the prevalence of SARS-CoV-2. Understanding the technical limitations of diagnostic testing for individual buildings would help inform future surveillance programs. We report our diagnostic and clinical data monitoring of buildings on a college campus in New York City during the spring 2021 semester. Frequent nasal testing, mitigation measures, and public health protocols provided a context in which to study the effectiveness of wastewater-based epidemiology. Our efforts could not consistently detect individual positive COVID-19 cases, but sensitivity is significantly improved in detecting two simultaneous cases. We therefore contend that wastewater surveillance may be more practically suited for the mitigation of outbreak clusters.

## INTRODUCTION

The COVID-19 pandemic fueled a resurgence of wastewater-based epidemiology to monitor pathogen levels in large-scale sewersheds. Measurement of viral shedding in wastewater has long been practiced to detect a variety of targets. The public health need to understand the dynamics of COVID-19 spread led to adaptation of existing methods to SARS-CoV-2 (1–3). Initial efforts demonstrated the feasibility of detection at the scale of entire metropolitan areas (4–6). An early hope that wastewater surveillance could forecast future trends in clinical cases was realized upon comparison with clinical data (7, 8).

The need for epidemiological surveillance also utilized wastewater monitoring in small catchment areas. College campuses, because of their essential function and inherent need for residential occupancy, were particularly active in such efforts (9). The potential of testing an essentially large pooled sample of many people at one time without waiting for a symptomatic case to present at the clinic could be valuable in terms of both efficiency and cost. The scale of implementation, however, generates challenges not found at the level of entire cities. Building-resolution wastewater surveillance may be limited by complications of population size and water flow. The size of dormitories can vary widely, but given that many dormitories house only ~100 residents, the limit of detection for sampling methodologies is uncertain. A positive case must, in order to be detected, use a bathroom in a small temporal window during which the shed virus can be captured. What then, is the practical limit at which wastewater surveillance remains effective?

We present our assessment of a wastewater surveillance program at Barnard College, a small residential liberal arts school in New York City, over the course of the first semester in which students returned to campus housing in large numbers. Wastewater testing results were compared to a parallel program of asymptomatic nasal testing. The COVID-19 case burden was low enough such that cases in campus buildings would mostly appear one at a time, yet there were enough cases such that the sample size yielded meaningful assessment statistics. These conditions allowed determination of surveillance effectiveness at the threshold of detection. Our program is also readily adaptable to monitoring emerging variants of concern.

## RESULTS

### Feasibility of wastewater surveillance.

We monitored the isolation building into which positive cases were moved (Table 1). Comparison of SARS-CoV-2 levels inferred from PCR cycle threshold (*C_T_*) values did not correlate with the number of students in the building, which ranged from 0 to 6 (Table 2). This building, however, had very few inhabitants. Given the low flow of water, no correlation was expected. The only residents were positive cases who contributed all the wastewater. A new isolated student added both more water and more waste containing viral particles. Thus, adding more positive cases may not have altered the observed concentration if cases shed similar amounts. Examining the data, it was also clear that concentrations can vary widely. For example, the highest detected shedding occurred with only one student in isolation (Table 2). Lacking correlation between *C_T_* values and positive case numbers, we therefore treated all positive results similarly when comparing wastewater surveillance with nasal testing. For data analysis, we chose to categorize the wastewater testing results as either positive or negative rather than incorporate continuous *C_T_* values.

**TABLE 1:** Details of urban college buildings monitored in SARS-CoV-2 wastewater-based epidemiology program

Building	Occupants (no.)^*a*^		Establishments (no.)^*b*^		Sampling schedule		
	Students	Nonstudents	Restaurants	Shops	Collection^*d*^	Time	*n*/h
Dormitory 1	138	1	0	0	M, W, F	0900^*c*^−0900	1
Dormitory 2	188	4	2	1	M, W, F	2100^*c*^−0900	2
Isolation	Fluctuated	0	0	0	M, W, F	0900^*c*^−0900	1

a Students participated in the campus testing and isolation protocol that included twice-weekly nasal testing and 10-day isolation period for positive cases. Nonstudents did not participate in the same protocols.

b Non-college-affiliated businesses that shared the same sewage outflow pipe. Employees did not participate in the campus testing and isolation protocol.

c Sampling start time began on the day prior to collection.

d M, W, F, Monday, Wednesday, Friday, respectively.

**TABLE 2:** Detection of SARS-CoV-2 in isolation building wastewater by reverse transcription-quantitative PCR

Sample (no. of positive cases, building, and date [mo and day])	Undiluted RNA (*C_T_*)		1:10 dilution of RNA (*C_T_*)	
	Replicate 1^*a*^	Replicate 2^*a*^	Replicate 1^*b*^	Replicate 2^*b*^
6 cases				
Isolation, Jan. 26	36.2	35.9	ND^*c*^	36.7
5 cases				
Isolation, Jan. 20	40^*d*^	40	36.7	35.3
Isolation, Jan. 22	37.4	36.7	36.9	38.2
4 cases				
Isolation, Mar. 29	40	ND	40	ND
3 cases				
Isolation, Jan. 29	39.0	ND	ND	ND
Isolation, Feb. 3	34.1	35.1	37.8	35.8
Isolation, Mar. 22	33.9	33.9	37.2	36.0
Isolation, Mar. 31	36.3	36.5	ND	38.0
2 cases				
Isolation, Jan. 18	32.2	32.3	31.8	32.8
Isolation, Apr. 2	36.2	39.2	35.4	36.7
1 case				
Isolation, Mar. 5	ND	37.2	40	ND
Isolation, Mar. 8	37.9	37.8	ND	ND
Isolation, Mar. 10	35.1	34.5	35.3	36.2
Isolation, Mar. 12	35.0	34.8	35.4	34.8
Isolation, Mar. 15	38.0	ND	ND	ND
Isolation, Mar. 17	ND	37.3	ND	ND
Isolation, Apr. 5	40	39.3	37.9	39.5
Isolation, Apr. 9	ND	ND	39.5	ND
Isolation, Apr. 19	39.2	37.3	ND	ND
Isolation, Apr. 23	37.0	37.0	ND	36.0
Isolation, Apr. 26	30.5	30.0	31.4	31.3
0 cases				
Isolation, Feb. 17	ND	37.5	ND	ND
Isolation, Feb. 19	ND	38.8	ND	ND

a Technical replicate measurements of the same biological replicate.

b Technical replicate measurements of the same biological replicate.

c ND, not detected.

d Positive signals in the last 5 of the 45 PCR cycles are assigned values of 40.

**FIG 1:**
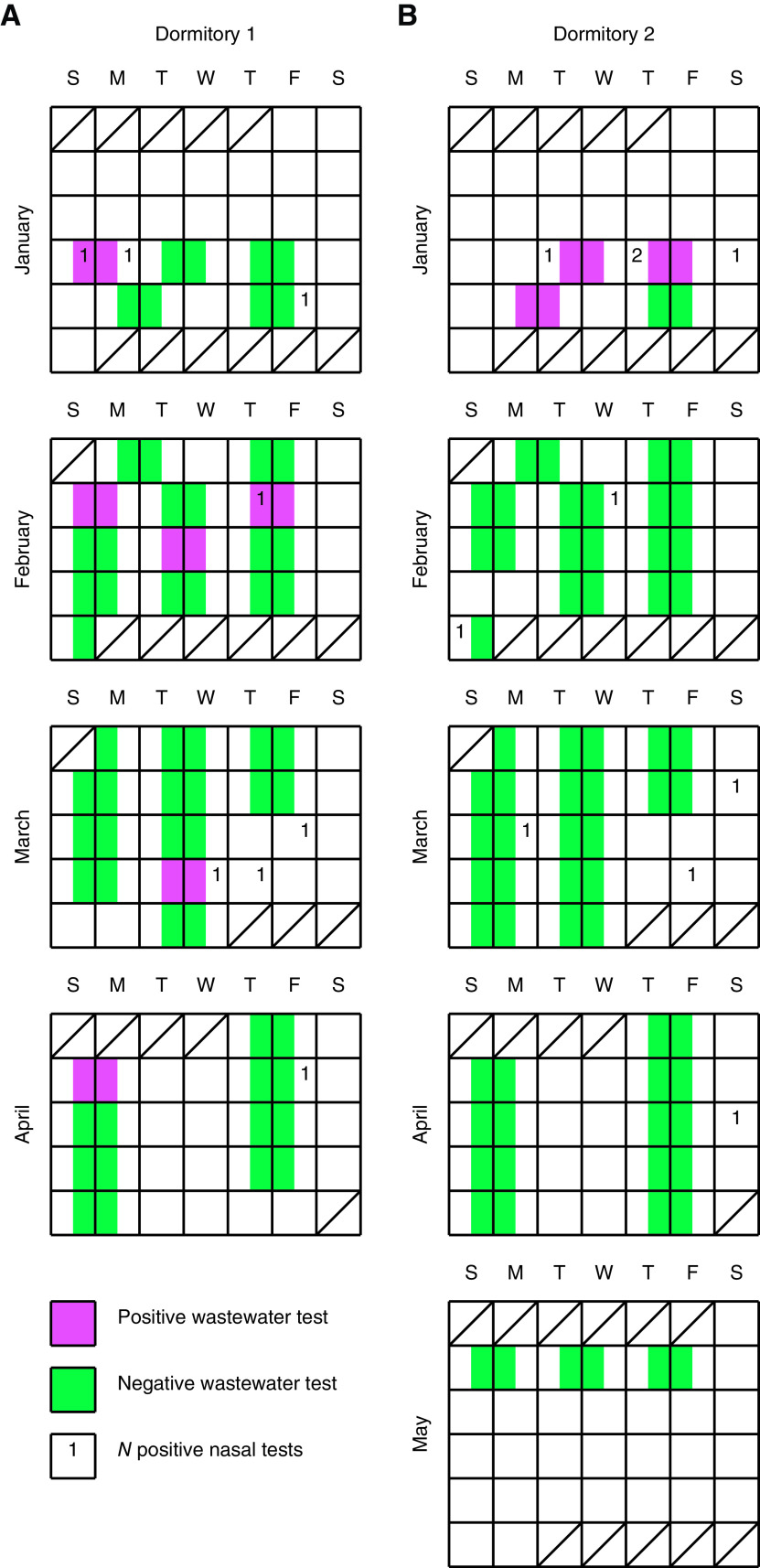
Combined wastewater and nasal diagnostic surveillance of SARS-CoV-2 in dormitory buildings. Dates of each test are shown in a calendar format from January to May 2021. Positive and negative wastewater tests are depicted as reddish purple and bluish green squares, respectively. The composite sampling period spans the overnight period between two calendar days. Positive nasal tests per day are depicted by numerals. The date indicated marks the time at which the positive case was moved out of the dormitory. Numeral placement inside or outside a colored square indicates that timing with the wastewater composite sampling period does or does not overlap, respectively. S, M, T, W, T, F, and S, Sunday, Monday, Tuesday, Wednesday, Thursday, Friday, and Saturday, respectively.

Two dormitories monitored over a semester indicated that positive cases identified by nasal swabs were usually associated with detection of SARS-CoV-2 in wastewater (Fig. 1 and Table 3). Quantitative assessment of the wastewater surveillance program was performed by comparing the results with asymptomatic nasal PCR testing (Table 4). Using the 2 dormitory buildings, diagnostic testing yielded a modest positive predictive power of 50 to 67% but a reliable negative predictive power of 83 to 90%. The assay demonstrated a low sensitivity of 25 to 50% but a high specificity of 90 to 97%. Analysis of aggregated data revealed similar results with a positive predictive power of 56%, negative predictive power of 86%, sensitivity of 36%, and specificity of 93%.

**TABLE 3:** Detection of SARS-CoV-2 in dormitory building wastewater by reverse transcription-quantitative PCR

Sample (building and date [mo and day])	Undiluted RNA (*C_T_*)		1:10 dilution of RNA (*C_T_*)	
	Replicate 1^*a*^	Replicate 2^*a*^	Replicate 1^*b*^	Replicate 2^*b*^
Dormitory 1, Jan. 18^*c*^	38.2	ND^*d*^	ND	ND
Dormitory 1, Feb. 8^*e*^	37.7	ND	ND	ND
Dormitory 1, Feb. 12^*f*^	39.1	38.2	ND	ND
Dormitory 1, Feb. 17^*e*^	37.9	ND	ND	ND
Dormitory 1, Mar. 24^*c*^	ND	ND	37.2	ND
Dormitory 1, Apr. 5^*e*^	ND	ND	ND	38.8
Dormitory 2, Jan. 20^*c*^	ND	35.4	ND	ND
Dormitory 2, Jan. 22^*f*^	36.3	35.6	36.7	35.0
Dormitory 2, Jan. 26^*e*^	37.4	39.2	ND	ND

a Technical replicate measurements of the same biological replicate.

b Technical replicate measurements of the same biological replicate.

c True positive, 2 cases in building.

d ND, not detected.

e False positive.

f True positive, 1 case in building.

**TABLE 4:** Predictive power, specificity, and sensitivity of wastewater-based SARS-CoV-2 surveillance calculated by comparison to concurrent asymptomatic nasal PCR testing of building occupants

Samples (no. of positive cases and building[s])	True positives (no.)	False positives (no.)	True negatives (no.)	False negatives (no.)	Positive predictive power (%)	Negative predictive power (%)	Sensitivity (%)	Specificity (%)
All cases^*a*^								
Dormitory 1	3	3	26	3	50	90	50	90
Dormitory 2	2	1	29	6	67	83	25	97
All Dormitories	5	4	55	9	56	86	36	93
1 case^*b*^								
All Dormitories	2	NA^*d*^	NA	9	NA	NA	18	NA
2 cases^*c*^								
All Dormitories	3	NA	NA	0	NA	NA	100	NA

a Considers all wastewater tests regardless of the number of positive cases identified by asymptomatic nasal testing.

b Considers only wastewater tests followed by 1 positive case identified by asymptomatic nasal testing.

c Considers only wastewater tests followed by 2 positive cases identified by asymptomatic nasal testing.

d NA, not applicable.

### Sensitivity of detection.

To probe sensitivity near a potential limit of detection, we asked whether or not the number of simultaneous positive cases affects our ability to detect SARS-CoV-2 in wastewater. Overall sensitivity was low, yet we observed that wastewater testing of buildings containing more than one positive case appeared reliable. The data from the semester were thus stratified by number of positive cases in 1 wastewater testing interval. To achieve a reasonable sample size, the data were aggregated between the 2 buildings. A two-tailed Fisher exact test rejects the null hypothesis that a single positive case and two positive cases are equally likely to be detected (*P *= 0.0275), with the qualifier that *n* is modest. Our program therefore has significantly higher sensitivity for detecting simultaneous positive cases in a building than for detecting a single positive case.

### Tracking SARS-CoV-2 variants.

**FIG 2:**
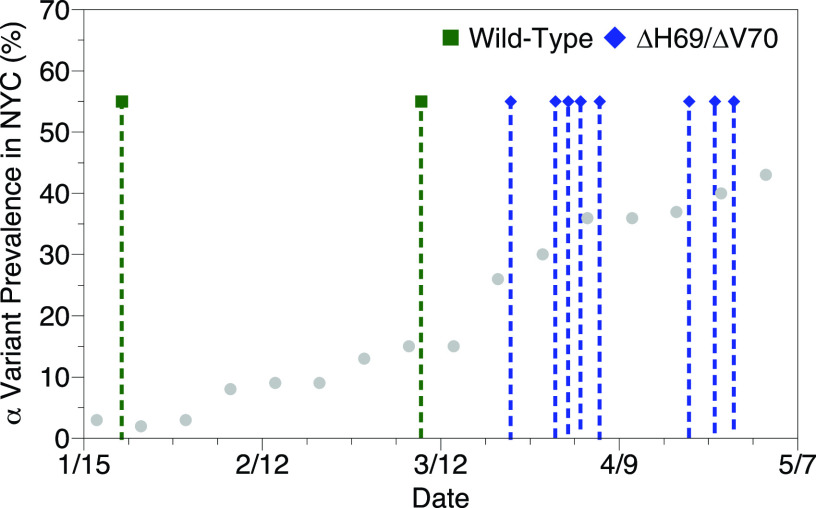
Mutation-specific detection of SARS-CoV-2 variants on campus during the spread of the α variant in New York City (NYC). The prevalence of the α variant based on genomic sequencing is plotted against the calendar date. Positive tests from mutation-specific PCR assays are depicted by symbols with drop lines at the top. Detections of the wild-type sequence and ΔH69/ΔV70 mutation are depicted by green squares and blue diamonds, respectively.

We also asked whether building-level surveillance could detect the arrival of an emerging variant of concern on campus to better inform a public health response. The ΔH69/ΔV70 spike protein mutation can be found in the α variant that spread through the United States in early 2021 but in not wild-type SARS-CoV-2 (10). Not all wastewater samples positive using the CDC N1 primers and probe were also positive with the allele-specific set, likely due to different amplification sensitivity. Two samples, one from January and one from early March, were positive for the wild-type sequence. Sparse positive cases in that time frame precluded more dense genotyping. Nine samples, from late March onward, were positive for the ΔH69/ΔV70 mutation. None of our samples contained a mixture of the two sequences. During this time frame, the prevalence of the α variant greatly increased in New York City as shown by variant sequencing conducted by the Department of Health and Mental Hygiene. On campus, the transition between the wild-type and ΔH69/ΔV70 sequences coincided with the spread of the α variant throughout New York City (Fig. 2). We interpret this result as the spread of the α variant from New York City to campus.

## DISCUSSION

### Wastewater-based epidemiology of college campuses.

Our team established a successful SARS-CoV-2 wastewater surveillance program on the campus of a small residential liberal arts college in the middle of New York City during the COVID-19 pandemic. Detection of virus was certainly feasible with the sampling protocol and diagnostic testing chosen. A well-organized public health response allowed us to compare results from wastewater surveillance with those of frequent asymptomatic nasal testing. The rapid identification of positive cases with asymptomatic nasal testing twice a week followed by timely transfer to an isolation building generated particular confidence in classifying true-positive and true-negative results determined by wastewater surveillance. In the fall 2021 semester, students were not moved to a dedicated isolation building and overlap with new positive cases prevented similar data analysis (data not shown). The program was also adaptable to measuring the appearance of a variant of concern.

We want to emphasize key caveats to our work regarding the impact of mitigation measures on wastewater surveillance. First, the context of the return to campus in spring 2021 was very controlled compared to current real-world scenarios. We suspect that strict building access policies and testing frequencies in particular improve the statistics of wastewater surveillance. Prevention of guests from using bathrooms likely reduces the false-positive number. Frequent surveillance with twice-a-week nasal testing generates confidence in the true-negative number. Second, our study was performed prior to the wide availability of vaccines. Because shedding is reduced in vaccinated individuals (11, 12), detection of positive cases may be more difficult in a population with up-to-date immunizations. Third, we caution that our sample size is modest and yet also represents a difficult-to-find study that directly compares testing of individual nasal swabs with pooled building wastewater near the limit of sensitivity. Statistical analysis with an exact significance test appropriate for small sample sizes argues that an inference may be made. Taken conservatively, our study at least suggests a trend where effectiveness of surveillance increases when attempting to detect more than one positive case.

### Practical limit of detection for individual buildings.

Our assessment contributes to the body of literature on building-resolution SARS-CoV-2 surveillance by uncovering a practical limit of detection. Many campus studies did not address this specific issue. Surge testing, the practice of using a positive wastewater test to compel subsequent testing of all individuals in a building, does indeed identify positive cases (13–15), but lack of concurrent surveillance makes understanding of detection limits incomplete. Surge testing can measure positive predictive power but not negative predictive power, specificity, nor sensitivity. Other studies may have obtained data to query the detection threshold (16–21) but did not report the analysis. One campus program reported ~10% sensitivity for detecting one or two sporadic cases in a residence hall (22). Because of a coincidental coalescence of multiple factors, our campus generally experienced one positive case at a time occurring in a building, and we choose to focus on the question of determining a limit of detection.

Our own program’s effectiveness at detecting individual cases was reasonable but mixed. The overall predictive power of ~60% and sensitivity of ~40% speak to limitations. Perhaps this should not be unexpected because multiple criteria need to be met for a positive wastewater signal to emerge. Even with composite sampling, which we did at 1-h or 30-minute intervals, the vast majority of wastewater may flow out of a building without being collected. Not all positive cases shed virus into feces (23, 24). Even if a sample were collected at the necessary time of deposition, a positive case must also be shedding sufficient virus to be discovered by our assay.

The sensitivity of our wastewater surveillance greatly increases, however, with more than one positive case in a building. Although our sample size with two simultaneous cases is limited, the difference in sensitivity is significant. This appears to be the threshold for consistent detection with our assay conditions. In our experience, building-resolution surveillance may not be ideal at uncovering single positive cases. The demonstrated abilities to reliably detect multiple positive cases as well as identify variants of concern combine into a potentially effective and informative surveillance program. This cost-effective approach may serve practical utility if frequent individual testing is not sustainable. Wastewater-based epidemiology could be realistically used to detect and characterize SARS-CoV-2 outbreak clusters consisting of potentially as few as two cases.

## MATERIALS AND METHODS

### Surveillance buildings.

Monitoring was performed in 3 buildings over the course of the spring 2021 semester during which students returned to campus for the first time in large numbers during the COVID-19 pandemic. One isolation building and 2 residential dormitories generated the data for this study (Table 1). Composite sampling was scheduled over a 24-h period when feasible to maximize the possibility for detecting shed virus. In the 1 building with 2 restaurants connected to the same sewage outflow line, composite sampling was reduced to a 12-h period to avoid the bulk of the food service business hours.

### Wastewater sampling.

For composite sampling of each building, we installed a Sentry ultracompact water sampler (N-Con Systems, Arnoldsville, GA) in the U-bend of the outflow pipe that connected directly to the New York City sewer system. Wastewater samples included water from toilets in addition to gray water from sinks, showers, kitchens, laundry machines, and roof runoff during rain or snow. In dormitory 1 and the isolation building, both of which performed only residential functions, the autosampler collected ~50 mL of wastewater once an hour for 24 h from 9:00 a.m. to 9:00 a.m. the next day. To avoid sampling during hours of operation of businesses in dormitory 2, the autosampler collected ~50 mL of wastewater twice an hour every hour for 12 h from 9:00 p.m. to 9:00 a.m. the next day. Composite samples were kept at 4°C in an undercounter refrigerator until collection and then transported to the laboratory in an insulated cooler. Wastewater sampling was occasionally disturbed by weather, holidays, or technical errors but otherwise generally adhered to the planned schedule (Table 1). Sampling frequency was decreased from three times a week to twice a week at the end of the semester because of low positive case counts.

### Wastewater processing and diagnostic testing.

Wastewater was prepared, RNA was purified, and PCR was performed according to our published protocol for accessible processing of SARS-CoV-2 from wastewater (25). An undiluted solution and a 1:10-diluted RNA solution, both in technical duplicate, were tested for each sample. A positive N1 signal was indicated by a *C_T_* value of <40 in any of the four PCRs.

### Asymptomatic nasal testing and public health protocols.

An independent asymptomatic testing program was established at Barnard College using the Broad Institute Safe for School Program. Students, staff, and faculty tested at different frequencies. During the spring 2021 academic semester, residential and nonresidential students were required to participate in twice-weekly asymptomatic nasal PCR testing. Compliant scheduling generally occurred with 3 or 4 days in between tests. Staff and faculty who interacted with students were tested weekly while those who did not directly interact with students were tested monthly. If a student tested positive, that case was moved to a dedicated isolation building within ~2 h of notification of positive nasal PCR test results by the Barnard College pandemic response team. Positive cases isolated for 10 days per Centers for Disease Control and Prevention and New York State guidelines at that time. Throughout the semester, students could access only their own dormitories and external guests were not allowed to visit.

### Comparison of wastewater and nasal testing.

Wastewater test results were compared to the positive cases identified by nasal tests in the time interval from the start of the composite sampling period until the start of the next composite sampling period. The phone call during which a student was contacted by the Barnard College pandemic response team to coordinate movement to the isolation building was used as our timestamp for overlap with wastewater testing. This marked the last approximate time in which that positive test could shed detectable SARS-CoV-2 into wastewater. We classified a positive wastewater test as a true positive if a positive case in the building was identified with nasal testing. We classified a negative wastewater test as a true negative if no positive cases in the building were identified with nasal testing. Positive predictive power, negative predictive power, sensitivity, and specificity were calculated from ratios of true positives, false positives, true negatives, and false negatives (18).

### SARS-CoV-2 variant detection.

RNA from samples collected and stored at −80°C between January 2021 and April 2021 was reanalyzed for presence of the B.1.1.7 α variant. For PCR testing, we used primers specific for either the wild-type sequence of SARS-CoV-2 or the Δ69/Δ70 spike protein deletion as packaged in the S.delH69V70 TaqMan SARS-CoV-2 mutation panel assay (Thermo Fisher Scientific, Waltham, MA) (26). We used 0.5 μL of the 40× stock solution per 20-μL reaction mixture in our adaptation of the CDC PCR assay (25). This deletion within the S protein found in the B.1.1.7 α variant is also found in the B.1.525 η variant, but this variant did not spread significantly in New York City. Because the α variant emerged in New York City in a background predominantly consisting of the original wild-type sequence, we interpret signals positive for the Δ69/Δ70 deletion as indicating the appearance of the α variant. New York City variant data were accessed from the New York City Department of Health and Mental Hygiene GitHub (https://github.com/nychealth/coronavirus-data/tree/master/variants). Cumulative percentages were determined using data from sequenced SARS-CoV-2 infections across a 4-week period.

### Human subject research.

Our work includes the collection/study of data or specimens recorded such that subjects cannot be identified. This project was determined to be exempt from review by the Barnard College Institutional Review Board.
